# Aqua­{*N*-[(4-methyl­phen­yl)sulfon­yl]glycinato(2−)-κ^2^ *N*,*O*}(1,10-phenan­throline)copper(II)

**DOI:** 10.1107/S1600536810036135

**Published:** 2010-09-15

**Authors:** Miao-Ling Huang

**Affiliations:** a Department of Chemistry and Life Science, Quanzhou Normal University, Fujian 362000, People’s Republic of China

## Abstract

In the title complex, [Cu(C_9_H_9_NO_4_S)(C_12_H_8_N_2_)(H_2_O)], the Cu^II^ ion is coordinated in a distorted square-pyramidal geometry by the two N atoms from a 1,10-phenanthroline ligand, one N atom from the deprotonated amino group of an *N*-tosyl­glycinate ligand, one O atom from the carboxyl­ate part of the *N*-tosyl­glycinate ligand and a water O atom. Inter­molecular O—H⋯O hydrogen bonds involving the water H atoms link neighboring mol­ecules into supra­molecular chains along [010]. Weak π–π stacking inter­actions [centroid–centroid distances of 3.456 (1) and 3.691 (1) Å] between the benzene rings of 1,10-phenanthroline ligands of adjacent mol­ecules extend the chains into a layer structure parallel to (001).

## Related literature

For the coordination chemistry of *N*-sulfonyl amino acids, see: Liang *et al.* (2004 ▶); Ma *et al.* (2008 ▶). For related structures, see: Battaglia *et al.* (1983 ▶); Antolini *et al.* (1985 ▶); Menabue & Saladini (1991 ▶).
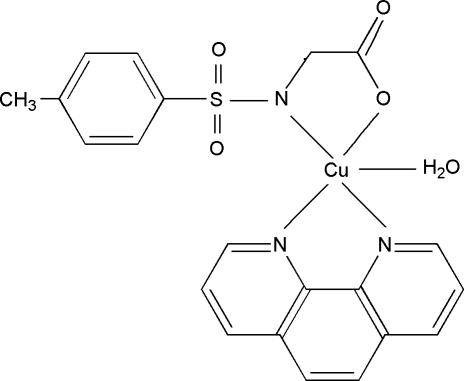


## Experimental

### 

#### Crystal data

[Cu(C_9_H_9_NO_4_S)(C_12_H_8_N_2_)(H_2_O)]
*M* *_r_* = 488.99
Monoclinic, 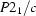

*a* = 14.0788 (11) Å
*b* = 7.0588 (6) Å
*c* = 20.6993 (17) Å
β = 103.826 (1)°
*V* = 1997.5 (3) Å^3^
*Z* = 4
Mo *K*α radiation
μ = 1.24 mm^−1^
*T* = 296 K
0.32 × 0.29 × 0.25 mm

#### Data collection

Bruker SMART CCD area-detector diffractometer
Absorption correction: multi-scan (*SADABS*; Sheldrick, 2003 ▶) *T*_min_ = 0.693, *T*_max_ = 0.747
14609 measured reflections
3713 independent reflections
3341 reflections with *I* > 2σ(*I*)
*R*_int_ = 0.019

#### Refinement

*R*[*F* ^2^ > 2σ(*F* ^2^)] = 0.024
*wR*(*F* ^2^) = 0.065
*S* = 1.06
3713 reflections
281 parameters
H-atom parameters constrained
Δρ_max_ = 0.27 e Å^−3^
Δρ_min_ = −0.30 e Å^−3^

### 

Data collection: *SMART* (Bruker, 2001 ▶); cell refinement: *SAINT* (Bruker, 2003 ▶); data reduction: *SAINT*; program(s) used to solve structure: *SHELXS97* (Sheldrick, 2008 ▶); program(s) used to refine structure: *SHELXL97* (Sheldrick, 2008 ▶); molecular graphics: *SHELXTL* (Sheldrick, 2008 ▶); software used to prepare material for publication: *SHELXTL*.

## Supplementary Material

Crystal structure: contains datablocks global, I. DOI: 10.1107/S1600536810036135/zq2057sup1.cif

Structure factors: contains datablocks I. DOI: 10.1107/S1600536810036135/zq2057Isup2.hkl

Additional supplementary materials:  crystallographic information; 3D view; checkCIF report

## Figures and Tables

**Table 1:** Hydrogen-bond geometry (Å, °)

*D*—H⋯*A*	*D*—H	H⋯*A*	*D*⋯*A*	*D*—H⋯*A*
O5—H1*W*⋯O2^i^	0.85	1.87	2.717 (2)	175
O5—H2*W*⋯O4^ii^	0.85	2.00	2.847 (2)	174

Symmetry codes: (i) 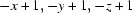; (ii) 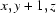.

**Fig. 1.**
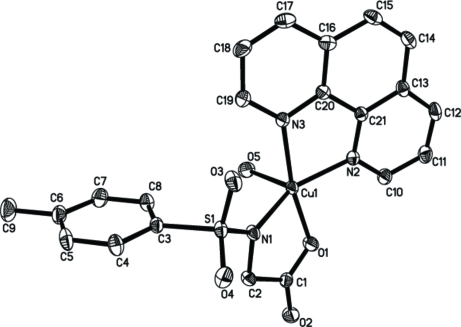
The molecular structure of the title compound. Displacement ellipsoids are drawn at the 30% probability level. All hydrogen atoms have been omitted for clarity reasons.

**Fig. 2.**
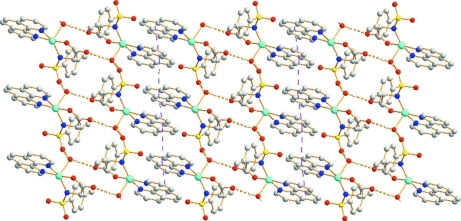
Projection showing the two-dimensional structure of the title compound formed by the intermolecular hydrogen bonds and the π-π stacking interactions.

## supplementary crystallographic information

### Comment

As a kind of amino acid derivatives, the N-protected amino acid plays an important role in participating in the process of the life activity. The substitution of an Ar—SO_2_-group on the amine nitrogen of amino acids, such as glycine and B-alanine, increases the coordination donors behavior of amino acids to three types of O, N donors from carboxyl, sulfoxyl and amine respectively, which may lead to different coordination modes, thus is of great interest in studying the coordination chemistry of *N*-sulfonyl- amino acids for many chemical workers (Ma *et al.*, 2008; Liang *et al.*, 2004; Battaglia *et al.*, 1983; Menabue *et al.*, 1991; Antolini *et al.*, 1985). In order to continue the research, we synthesized the title complex [Cu(C_9_H_9_NO_4_S)(C_12_H_8_N_2_)(H_2_O)] and characterized it by an elemental analysis and a single-crystal X-ray diffraction analysis.

The molecular structure and crystal packing diagram of the title compound are presented in Figs. 1 and 2, respectively. The asymmetric unit contains one copper cation, one Ts-gly anion, one phen molecule and one coordinated water molecule. The central copper ion adopts a distorted square-pyramidal geometry by two N(N2, N3) atoms of the 1,10-phenanthroline ligand, one N(N1) and one O(O1) atoms of the Ts-gly ion occupying basal site, while the apical position is occupied by another O atom of a water molecule. The Cu—O1 bond distance of 1.9269 (13) Å is shorter than those of other N-protected glycine complexes (1.933–1.967 Å) [Battaglia, *et al.*, 1983; Antolini, *et al.*, 1985; Menabue & Saladini, 1991]. Furthermore, the C—O bond distance for the coordinated O atom (1.282 (2) Å) is significantly longer than that for the uncoordinated O atom (1.233 (2) Å), which is similar to previously reported complexes (Battaglia, *et al.*, 1983; Antolini, *et al.*, 1985).

Intermolecular hydrogen bonds involving the water H atoms, *O*(5)—H(1W)···O(2)^i^, O(5)—H(2W)···O(4)^ii ^(Table 1), link the neighboring molecules into one-dimensional supramolecular chains. Weak π-π stacking interactions between benzene rings of 1,10-phenanthroline ligands from adjacent molecules (centroid distances of 3.456Å and 3.691 Å) extend the one-dimensional chains into a two-dimensional layer structure.

### Experimental

To a solution of Ts-gly (1 mmol) in water-DMF 1:1 (10 ml), an aqueous solution (5 ml) of CuCl_2_.2H_2_O (1 mmol) and a solution of 1,10-phenanthroline (1 mmol) in ethanol (95%, 5 ml) was added. After refluxing for 12 h at 343 K, the mixture was filtered off while hot. The green single crystals suitable for a X-ray analysis were obtained by slow evaporation of the filtrate at room temperature after 41 days. IR(KBr): 3442(*vs*), 1638(*vs*), 1586(*s*), 1518(*s*), 1493(*m*), 1434(*s*), 1382(*vs*), 1348(*m*), 1319(*m*), 1243(*vs*), 1132(*vs*), 1112(*vs*), 1078(*s*), 1007(*s*), 967(*s*), 940(*m*), 847(*s*), 820(*m*), 723(*s*),663(*s*), 589(*s*), 545(*m*) cm^-1^.

### Refinement

H atoms bonded to C were placed geometrically and treated as riding with C—H = 0.93–0.97 Å and *U*_iso_(H) = 1.2*U*_eq_(C). The water H atoms were found in difference Fourier maps and refined with O—H = 0.85 Å and *U*_iso_(H) = 1.5*U*_eq_(O).

### Crystal data

**Table:**

[Cu(C_9_H_9_NO_4_S)(C_12_H_8_N_2_)(H_2_O)]	*F*(000) = 1004
*M**_r_* = 488.99	*D*_x_ = 1.626 Mg m^−^^3^
Monoclinic, *P*2_1_/*c*	Mo *K*α radiation, λ = 0.71073 Å
*a* = 14.0788 (11) Å	Cell parameters from 7260 reflections
*b* = 7.0588 (6) Å	θ = 2.8–28.2°
*c* = 20.6993 (17) Å	µ = 1.24 mm^−^^1^
β = 103.826 (1)°	*T* = 296 K
*V* = 1997.5 (3) Å^3^	Block, green
*Z* = 4	0.32 × 0.29 × 0.25 mm

### Data collection

**Table:**

Bruker SMART CCD area-detector diffractometer	3713 independent reflections
Radiation source: fine-focus sealed tube	3341 reflections with *I* > 2σ(*I*)
graphite	*R*_int_ = 0.019
φ and ω scans	θ_max_ = 25.5°, θ_min_ = 2.8°
Absorption correction: multi-scan (*SADABS*; Sheldrick, 2003)	*h* = −17→16
*T*_min_ = 0.693, *T*_max_ = 0.747	*k* = −8→8
14609 measured reflections	*l* = −25→25

### Refinement

**Table:**

Refinement on *F*^2^	Primary atom site location: structure-invariant direct methods
Least-squares matrix: full	Secondary atom site location: difference Fourier map
*R*[*F*^2^ > 2σ(*F*^2^)] = 0.024	Hydrogen site location: inferred from neighbouring sites
*wR*(*F*^2^) = 0.065	H-atom parameters constrained
*S* = 1.06	*w* = 1/[σ^2^(*F*_o_^2^) + (0.0287*P*)^2^ + 1.2801*P*] where *P* = (*F*_o_^2^ + 2*F*_c_^2^)/3
3713 reflections	(Δ/σ)_max_ = 0.001
281 parameters	Δρ_max_ = 0.27 e Å^−^^3^
0 restraints	Δρ_min_ = −0.30 e Å^−^^3^

### Special details

**Table:**

Geometry. All e.s.d.'s (except the e.s.d. in the dihedral angle between two l.s. planes)are estimated using the full covariance matrix. The cell e.s.d.'s are takeninto account individually in the estimation of e.s.d.'s in distances, anglesand torsion angles; correlations between e.s.d.'s in cell parameters are onlyused when they are defined by crystal symmetry. An approximate (isotropic)treatment of cell e.s.d.'s is used for estimating e.s.d.'s involving l.s. planes.
Refinement. Refinement of *F*^2^ against ALL reflections. The weighted *R*-factor *wR* and goodness of fit *S* are based on *F*^2^, conventional *R*-factors *R* are based on *F*, with *F* set to zero for negative *F*^2^. The threshold expression of *F*^2^ > σ(*F*^2^) is used only for calculating *R*-factors(gt) *etc*. and is not relevant to the choice of reflections for refinement. *R*-factors based on *F*^2^ are statistically about twice as large as those based on *F*, and *R*- factors based on ALL data will be even larger.

### Fractional atomic coordinates and isotropic or equivalent isotropic displacement parameters (Å^2^)

**Table:**

	*x*	*y*	*z*	*U*_iso_*/*U*_eq_	
Cu1	0.245571 (16)	0.54423 (3)	0.471642 (10)	0.02714 (8)	
S1	0.24891 (3)	0.25311 (7)	0.34767 (2)	0.02718 (12)	
O1	0.35227 (10)	0.4521 (2)	0.54106 (6)	0.0376 (3)	
O2	0.49352 (10)	0.2987 (2)	0.55995 (7)	0.0408 (4)	
O3	0.14652 (10)	0.3036 (2)	0.32547 (7)	0.0402 (4)	
O4	0.26947 (12)	0.0512 (2)	0.34674 (7)	0.0403 (4)	
O5	0.32853 (10)	0.8041 (2)	0.45785 (8)	0.0430 (4)	
H1W	0.3856	0.7738	0.4546	0.064*	
H2W	0.3065	0.8774	0.4251	0.064*	
N1	0.29113 (11)	0.3460 (2)	0.41754 (7)	0.0278 (4)	
N2	0.17697 (11)	0.6304 (2)	0.54314 (7)	0.0277 (3)	
N3	0.12245 (11)	0.6608 (2)	0.41228 (7)	0.0295 (4)	
C1	0.41586 (13)	0.3491 (3)	0.52244 (9)	0.0282 (4)	
C2	0.39262 (13)	0.2906 (3)	0.45023 (9)	0.0296 (4)	
H2A	0.4378	0.3513	0.4280	0.036*	
H2B	0.4000	0.1545	0.4470	0.036*	
C3	0.30947 (13)	0.3556 (3)	0.28982 (9)	0.0270 (4)	
C4	0.32266 (16)	0.2503 (3)	0.23598 (10)	0.0377 (5)	
H4	0.3025	0.1246	0.2312	0.045*	
C5	0.36591 (16)	0.3329 (4)	0.18939 (10)	0.0428 (5)	
H5A	0.3745	0.2615	0.1534	0.051*	
C6	0.39666 (15)	0.5203 (3)	0.19541 (10)	0.0398 (5)	
C7	0.38440 (16)	0.6220 (3)	0.25016 (11)	0.0417 (5)	
H7	0.4056	0.7471	0.2554	0.050*	
C8	0.34130 (15)	0.5416 (3)	0.29733 (10)	0.0354 (5)	
H8	0.3339	0.6122	0.3337	0.042*	
C9	0.4445 (2)	0.6079 (5)	0.14447 (13)	0.0597 (7)	
H9A	0.4000	0.6037	0.1014	0.090*	
H9B	0.5027	0.5383	0.1433	0.090*	
H9C	0.4613	0.7372	0.1564	0.090*	
C10	0.20889 (15)	0.6202 (3)	0.60898 (9)	0.0345 (5)	
H10	0.2727	0.5803	0.6270	0.041*	
C11	0.14933 (17)	0.6676 (3)	0.65179 (10)	0.0407 (5)	
H11	0.1738	0.6595	0.6976	0.049*	
C12	0.05523 (16)	0.7258 (3)	0.62636 (11)	0.0376 (5)	
H12	0.0151	0.7554	0.6547	0.045*	
C13	0.01946 (14)	0.7407 (3)	0.55693 (10)	0.0304 (4)	
C14	−0.07680 (15)	0.8059 (3)	0.52450 (11)	0.0371 (5)	
H14	−0.1209	0.8361	0.5500	0.044*	
C15	−0.10434 (14)	0.8240 (3)	0.45780 (11)	0.0373 (5)	
H15	−0.1672	0.8662	0.4381	0.045*	
C16	−0.03842 (14)	0.7795 (3)	0.41629 (10)	0.0318 (4)	
C17	−0.05995 (16)	0.8052 (3)	0.34695 (11)	0.0405 (5)	
H17	−0.1213	0.8484	0.3243	0.049*	
C18	0.00994 (17)	0.7661 (4)	0.31331 (11)	0.0449 (6)	
H18	−0.0027	0.7878	0.2677	0.054*	
C19	0.10062 (16)	0.6933 (3)	0.34718 (10)	0.0394 (5)	
H19	0.1472	0.6667	0.3233	0.047*	
C20	0.05477 (13)	0.7091 (3)	0.44656 (9)	0.0265 (4)	
C21	0.08421 (13)	0.6912 (3)	0.51756 (9)	0.0255 (4)	

### Atomic displacement parameters (Å^2^)

**Table:**

	*U*^11^	*U*^22^	*U*^33^	*U*^12^	*U*^13^	*U*^23^
Cu1	0.02506 (13)	0.03777 (15)	0.01906 (12)	0.00538 (10)	0.00617 (9)	0.00056 (9)
S1	0.0249 (2)	0.0364 (3)	0.0212 (2)	−0.00450 (19)	0.00735 (18)	−0.00286 (19)
O1	0.0349 (8)	0.0548 (10)	0.0217 (7)	0.0140 (7)	0.0039 (6)	−0.0026 (6)
O2	0.0281 (7)	0.0573 (10)	0.0331 (8)	0.0079 (7)	0.0001 (6)	0.0035 (7)
O3	0.0231 (7)	0.0672 (11)	0.0296 (7)	−0.0047 (7)	0.0052 (6)	−0.0070 (7)
O4	0.0549 (9)	0.0337 (8)	0.0325 (8)	−0.0083 (7)	0.0112 (7)	−0.0030 (6)
O5	0.0338 (8)	0.0421 (9)	0.0539 (10)	−0.0004 (7)	0.0121 (7)	0.0114 (7)
N1	0.0222 (8)	0.0404 (10)	0.0206 (8)	0.0022 (7)	0.0048 (6)	−0.0032 (7)
N2	0.0301 (8)	0.0303 (9)	0.0240 (8)	0.0005 (7)	0.0087 (6)	−0.0003 (7)
N3	0.0291 (8)	0.0365 (9)	0.0230 (8)	0.0037 (7)	0.0068 (6)	0.0020 (7)
C1	0.0259 (10)	0.0330 (11)	0.0259 (9)	−0.0018 (8)	0.0065 (8)	0.0036 (8)
C2	0.0245 (9)	0.0353 (11)	0.0291 (10)	0.0014 (8)	0.0066 (8)	−0.0037 (8)
C3	0.0229 (9)	0.0372 (11)	0.0210 (9)	−0.0001 (8)	0.0056 (7)	−0.0006 (8)
C4	0.0428 (12)	0.0423 (12)	0.0310 (11)	−0.0082 (10)	0.0146 (9)	−0.0100 (9)
C5	0.0451 (13)	0.0600 (15)	0.0283 (11)	−0.0037 (11)	0.0185 (9)	−0.0091 (10)
C6	0.0317 (11)	0.0587 (15)	0.0313 (11)	−0.0005 (10)	0.0120 (9)	0.0079 (10)
C7	0.0460 (13)	0.0399 (12)	0.0413 (12)	−0.0065 (10)	0.0145 (10)	0.0042 (10)
C8	0.0403 (11)	0.0390 (12)	0.0296 (10)	−0.0014 (9)	0.0134 (9)	−0.0039 (9)
C9	0.0604 (16)	0.0773 (19)	0.0500 (15)	−0.0056 (14)	0.0299 (13)	0.0149 (14)
C10	0.0370 (11)	0.0415 (12)	0.0248 (10)	0.0012 (9)	0.0071 (8)	−0.0007 (9)
C11	0.0521 (13)	0.0482 (13)	0.0248 (10)	0.0024 (11)	0.0150 (9)	−0.0014 (9)
C12	0.0460 (13)	0.0381 (12)	0.0365 (11)	−0.0029 (10)	0.0252 (10)	−0.0042 (9)
C13	0.0338 (10)	0.0249 (10)	0.0369 (11)	−0.0049 (8)	0.0172 (9)	−0.0036 (8)
C14	0.0315 (11)	0.0321 (11)	0.0542 (14)	−0.0019 (9)	0.0233 (10)	−0.0034 (10)
C15	0.0228 (10)	0.0334 (11)	0.0560 (14)	0.0005 (8)	0.0098 (9)	−0.0008 (10)
C16	0.0266 (10)	0.0263 (10)	0.0406 (11)	−0.0018 (8)	0.0044 (8)	−0.0014 (8)
C17	0.0322 (11)	0.0408 (12)	0.0415 (12)	0.0042 (9)	−0.0051 (9)	0.0015 (10)
C18	0.0475 (13)	0.0553 (15)	0.0266 (11)	0.0079 (11)	−0.0017 (9)	0.0049 (10)
C19	0.0413 (12)	0.0509 (13)	0.0262 (10)	0.0083 (10)	0.0087 (9)	0.0037 (9)
C20	0.0263 (9)	0.0237 (9)	0.0299 (10)	−0.0018 (7)	0.0075 (8)	−0.0010 (8)
C21	0.0272 (9)	0.0225 (9)	0.0287 (9)	−0.0029 (7)	0.0101 (8)	−0.0017 (7)

### Geometric parameters (Å, °)

**Table:**

Cu1—O1	1.9269 (13)	C6—C9	1.512 (3)
Cu1—N1	1.9916 (16)	C7—C8	1.388 (3)
Cu1—N3	2.0429 (16)	C7—H7	0.9300
Cu1—N2	2.0435 (15)	C8—H8	0.9300
Cu1—O5	2.2296 (15)	C9—H9A	0.9600
S1—O3	1.4490 (14)	C9—H9B	0.9600
S1—O4	1.4557 (16)	C9—H9C	0.9600
S1—N1	1.5697 (15)	C10—C11	1.398 (3)
S1—C3	1.7812 (19)	C10—H10	0.9300
O1—C1	1.282 (2)	C11—C12	1.367 (3)
O2—C1	1.233 (2)	C11—H11	0.9300
O5—H1W	0.8499	C12—C13	1.409 (3)
O5—H2W	0.8500	C12—H12	0.9300
N1—C2	1.480 (2)	C13—C21	1.404 (3)
N2—C10	1.331 (2)	C13—C14	1.437 (3)
N2—C21	1.356 (2)	C14—C15	1.348 (3)
N3—C19	1.329 (2)	C14—H14	0.9300
N3—C20	1.360 (2)	C15—C16	1.442 (3)
C1—C2	1.509 (3)	C15—H15	0.9300
C2—H2A	0.9700	C16—C20	1.403 (3)
C2—H2B	0.9700	C16—C17	1.406 (3)
C3—C8	1.384 (3)	C17—C18	1.363 (3)
C3—C4	1.389 (3)	C17—H17	0.9300
C4—C5	1.386 (3)	C18—C19	1.399 (3)
C4—H4	0.9300	C18—H18	0.9300
C5—C6	1.388 (3)	C19—H19	0.9300
C5—H5A	0.9300	C20—C21	1.434 (3)
C6—C7	1.387 (3)		
			
O1—Cu1—N1	83.32 (6)	C5—C6—C9	120.5 (2)
O1—Cu1—N3	169.34 (6)	C6—C7—C8	121.6 (2)
N1—Cu1—N3	106.60 (6)	C6—C7—H7	119.2
O1—Cu1—N2	88.83 (6)	C8—C7—H7	119.2
N1—Cu1—N2	152.58 (7)	C3—C8—C7	119.55 (19)
N3—Cu1—N2	80.56 (6)	C3—C8—H8	120.2
O1—Cu1—O5	91.97 (6)	C7—C8—H8	120.2
N1—Cu1—O5	104.91 (6)	C6—C9—H9A	109.5
N3—Cu1—O5	89.24 (6)	C6—C9—H9B	109.5
N2—Cu1—O5	101.57 (6)	H9A—C9—H9B	109.5
O3—S1—O4	114.99 (9)	C6—C9—H9C	109.5
O3—S1—N1	108.57 (8)	H9A—C9—H9C	109.5
O4—S1—N1	112.86 (9)	H9B—C9—H9C	109.5
O3—S1—C3	106.66 (9)	N2—C10—C11	121.93 (19)
O4—S1—C3	105.09 (9)	N2—C10—H10	119.0
N1—S1—C3	108.24 (9)	C11—C10—H10	119.0
C1—O1—Cu1	116.31 (12)	C12—C11—C10	120.06 (19)
Cu1—O5—H1W	109.7	C12—C11—H11	120.0
Cu1—O5—H2W	120.0	C10—C11—H11	120.0
H1W—O5—H2W	105.2	C11—C12—C13	119.51 (18)
C2—N1—S1	114.99 (12)	C11—C12—H12	120.2
C2—N1—Cu1	109.52 (11)	C13—C12—H12	120.2
S1—N1—Cu1	135.20 (9)	C21—C13—C12	116.76 (18)
C10—N2—C21	118.35 (16)	C21—C13—C14	118.63 (18)
C10—N2—Cu1	128.54 (14)	C12—C13—C14	124.61 (18)
C21—N2—Cu1	112.89 (12)	C15—C14—C13	121.15 (19)
C19—N3—C20	117.69 (17)	C15—C14—H14	119.4
C19—N3—Cu1	129.57 (14)	C13—C14—H14	119.4
C20—N3—Cu1	112.74 (12)	C14—C15—C16	121.40 (19)
O2—C1—O1	123.55 (18)	C14—C15—H15	119.3
O2—C1—C2	119.62 (17)	C16—C15—H15	119.3
O1—C1—C2	116.82 (16)	C20—C16—C17	116.87 (18)
N1—C2—C1	109.72 (15)	C20—C16—C15	118.53 (19)
N1—C2—H2A	109.7	C17—C16—C15	124.58 (19)
C1—C2—H2A	109.7	C18—C17—C16	119.32 (19)
N1—C2—H2B	109.7	C18—C17—H17	120.3
C1—C2—H2B	109.7	C16—C17—H17	120.3
H2A—C2—H2B	108.2	C17—C18—C19	120.1 (2)
C8—C3—C4	119.75 (18)	C17—C18—H18	119.9
C8—C3—S1	120.27 (14)	C19—C18—H18	119.9
C4—C3—S1	119.96 (16)	N3—C19—C18	122.3 (2)
C5—C4—C3	119.9 (2)	N3—C19—H19	118.9
C5—C4—H4	120.1	C18—C19—H19	118.9
C3—C4—H4	120.1	N3—C20—C16	123.56 (17)
C4—C5—C6	121.2 (2)	N3—C20—C21	116.46 (16)
C4—C5—H5A	119.4	C16—C20—C21	119.94 (17)
C6—C5—H5A	119.4	N2—C21—C13	123.37 (17)
C7—C6—C5	118.00 (19)	N2—C21—C20	116.33 (16)
C7—C6—C9	121.4 (2)	C13—C21—C20	120.28 (17)
			
N1—Cu1—O1—C1	15.55 (14)	S1—C3—C4—C5	177.14 (16)
N3—Cu1—O1—C1	174.4 (3)	C3—C4—C5—C6	0.0 (3)
N2—Cu1—O1—C1	169.23 (15)	C4—C5—C6—C7	1.1 (3)
O5—Cu1—O1—C1	−89.23 (15)	C4—C5—C6—C9	179.5 (2)
O3—S1—N1—C2	173.74 (14)	C5—C6—C7—C8	−1.1 (3)
O4—S1—N1—C2	45.04 (16)	C9—C6—C7—C8	−179.4 (2)
C3—S1—N1—C2	−70.84 (16)	C4—C3—C8—C7	1.3 (3)
O3—S1—N1—Cu1	−13.30 (17)	S1—C3—C8—C7	−177.10 (16)
O4—S1—N1—Cu1	−142.00 (13)	C6—C7—C8—C3	−0.1 (3)
C3—S1—N1—Cu1	102.13 (14)	C21—N2—C10—C11	0.9 (3)
O1—Cu1—N1—C2	−18.70 (12)	Cu1—N2—C10—C11	−173.35 (16)
N3—Cu1—N1—C2	165.30 (12)	N2—C10—C11—C12	0.3 (3)
N2—Cu1—N1—C2	−93.00 (17)	C10—C11—C12—C13	−1.1 (3)
O5—Cu1—N1—C2	71.57 (13)	C11—C12—C13—C21	0.8 (3)
O1—Cu1—N1—S1	168.06 (15)	C11—C12—C13—C14	−177.9 (2)
N3—Cu1—N1—S1	−7.94 (16)	C21—C13—C14—C15	−1.6 (3)
N2—Cu1—N1—S1	93.76 (18)	C12—C13—C14—C15	177.1 (2)
O5—Cu1—N1—S1	−101.67 (14)	C13—C14—C15—C16	−0.2 (3)
O1—Cu1—N2—C10	2.40 (18)	C14—C15—C16—C20	2.4 (3)
N1—Cu1—N2—C10	75.4 (2)	C14—C15—C16—C17	−176.2 (2)
N3—Cu1—N2—C10	−176.63 (19)	C20—C16—C17—C18	−1.6 (3)
O5—Cu1—N2—C10	−89.37 (18)	C15—C16—C17—C18	177.0 (2)
O1—Cu1—N2—C21	−172.10 (14)	C16—C17—C18—C19	2.8 (4)
N1—Cu1—N2—C21	−99.09 (17)	C20—N3—C19—C18	−3.0 (3)
N3—Cu1—N2—C21	8.87 (13)	Cu1—N3—C19—C18	176.61 (17)
O5—Cu1—N2—C21	96.13 (13)	C17—C18—C19—N3	−0.4 (4)
O1—Cu1—N3—C19	166.8 (3)	C19—N3—C20—C16	4.2 (3)
N1—Cu1—N3—C19	−35.2 (2)	Cu1—N3—C20—C16	−175.48 (15)
N2—Cu1—N3—C19	172.0 (2)	C19—N3—C20—C21	−173.60 (18)
O5—Cu1—N3—C19	70.10 (19)	Cu1—N3—C20—C21	6.7 (2)
O1—Cu1—N3—C20	−13.6 (4)	C17—C16—C20—N3	−1.9 (3)
N1—Cu1—N3—C20	144.41 (13)	C15—C16—C20—N3	179.39 (18)
N2—Cu1—N3—C20	−8.39 (13)	C17—C16—C20—C21	175.84 (18)
O5—Cu1—N3—C20	−110.25 (13)	C15—C16—C20—C21	−2.9 (3)
Cu1—O1—C1—O2	171.25 (16)	C10—N2—C21—C13	−1.2 (3)
Cu1—O1—C1—C2	−8.1 (2)	Cu1—N2—C21—C13	173.90 (14)
S1—N1—C2—C1	−166.30 (13)	C10—N2—C21—C20	176.91 (17)
Cu1—N1—C2—C1	18.95 (19)	Cu1—N2—C21—C20	−8.0 (2)
O2—C1—C2—N1	172.71 (18)	C12—C13—C21—N2	0.4 (3)
O1—C1—C2—N1	−7.9 (2)	C14—C13—C21—N2	179.15 (18)
O3—S1—C3—C8	85.06 (17)	C12—C13—C21—C20	−177.67 (18)
O4—S1—C3—C8	−152.43 (16)	C14—C13—C21—C20	1.1 (3)
N1—S1—C3—C8	−31.60 (19)	N3—C20—C21—N2	0.9 (3)
O3—S1—C3—C4	−93.29 (18)	C16—C20—C21—N2	−177.04 (17)
O4—S1—C3—C4	29.22 (19)	N3—C20—C21—C13	179.05 (17)
N1—S1—C3—C4	150.05 (16)	C16—C20—C21—C13	1.2 (3)
C8—C3—C4—C5	−1.2 (3)		

### Hydrogen-bond geometry (Å, °)

**Table:**

*D*—H···*A*	*D*—H	H···*A*	*D*···*A*	*D*—H···*A*
O5—H1W···O2^i^	0.85	1.87	2.717 (2)	175
O5—H2W···O4^ii^	0.85	2.00	2.847 (2)	174

Symmetry codes: (i) −*x*+1, −*y*+1, −*z*+1; (ii) *x*, *y*+1, *z*.
